# Comparing the inflammatory profiles for incidence of diabetes mellitus and cardiovascular diseases: a prospective study exploring the ‘common soil’ hypothesis

**DOI:** 10.1186/s12933-018-0733-9

**Published:** 2018-06-12

**Authors:** Xue Bao, Yan Borné, Linda Johnson, Iram Faqir Muhammad, Margaretha Persson, Kaijun Niu, Gunnar Engström

**Affiliations:** 1 Nutritional Epidemiology Institute and School of Public Health, Tianjin Medical University, Tianjin, China; 2 Department of Clinical Sciences, Lund University, CRC 60:13, Jan Waldenströms gata 35, 20502 Malmö, Sweden

**Keywords:** Diabetes mellitus, Cardiovascular diseases, Cohort study, Inflammatory markers, Proportional hazards models, Survival analysis, Competing risks analysis

## Abstract

**Background:**

Chronic low-grade inflammation and associated insulin resistance and metabolic abnormalities have been proposed as ‘common soil’ for diabetes mellitus (DM) and cardiovascular disease (CVD). This paper aimed to investigate the inflammatory profiles of DM and CVD and to distinguish their shared and specific markers.

**Methods:**

Based on the Malmö Diet and Cancer cohort, total and differential leukocyte counts were measured in 25,969 participants without previous DM or CVD and were studied in relation to incident DM (mean follow-up 17.4 ± 5.58 years) and incident CVD (i.e., coronary events, including fatal and nonfatal myocardial infarction, or stroke); mean follow-up 17.7 ± 5.46 years, using multivariable Cox regression models. Furthermore, plasma concentrations of another seven inflammatory markers were examined in relation to incident DM and incident CVD in a sub-cohort of 4658 participants. The associations of each inflammatory marker with incident DM versus incident CVD were compared using the Lunn–McNeil competing risks approach. In sensitivity analyses, those who developed both DM and CVD during follow-up were excluded.

**Results:**

After adjustment for conventional risk factors, total and differential leukocyte counts, orosomucoid, and C-reactive protein were associated with an increased risk of both DM and CVD. Neutrophil to lymphocyte ratio, ceruloplasmin, alpha1-antitrypsin and soluble urokinase plasminogen activator receptor predicted increased risk of CVD but not DM, while haptoglobin and complement C3 showed the opposite pattern. In competing risks analyses, lymphocyte count and complement C3 had stronger associations with risk of DM than with risk of CVD (*p* for equal associations = 0.020 and 0.006). The reverse was true for neutrophil to lymphocyte ratio (*p* for equal associations = 0.025). Results were consistent in sensitivity analyses.

**Conclusions:**

The results indicated substantial similarities in the inflammatory profiles associated with DM and CVD. However, there are also significant differences. These findings may help discriminate between individuals at elevated risk of DM and those at elevated risk of CVD, which is a prerequisite for targeted therapies.

**Electronic supplementary material:**

The online version of this article (10.1186/s12933-018-0733-9) contains supplementary material, which is available to authorized users.

## Background

Diabetes mellitus (DM) is a well-known risk factor for cardiovascular disease (CVD) [1]. It has also been shown that CVD risk factors predict incident DM [2, 3]. The overlapping risk factors for DM and CVD support a hypothesis that the two diseases share common antecedents [4]. This view is further supported by shared molecular drivers, pathways, and gene subnetworks for DM and CVD that have recently been discovered using genetic and functional data from multiple human cohorts [5]. A vast overlap in gene expression alterations has also been identified in obesity-driven insulin resistance (IR) and atherosclerosis, which are critical pathogenic mechanisms underlying DM and CVD, respectively [6]. Moreover, many of these alterations are related to the inflammatory response pathway [6]. Chronic low-grade inflammation and associated IR and metabolic abnormalities thus constitute a possible ‘common soil’ for DM and CVD [7–9].

Though inflammation is firmly established as central to the development of both DM and CVD, its importance in the pathogenesis may differ between DM and CVD [9–11]. The specific inflammatory processes involved in DM versus CVD may differ as well [12–15]. In DM, enlarged adipocytes and altered secretion of adipokines play a significant role in promoting inflammation in adipose tissue [16]. Islet β-cell dysfunction and IR, induced by inflammation, are involved in the pathogenesis of DM [6, 7]. Other inflammation-related mechanisms, such as endothelial dysfunction, atherosclerosis and increased plaque vulnerability, play important roles in CVD [6, 8]. For these reasons, it is possible that the inflammatory profiles are different for DM and CVD.

A wide range of inflammatory markers have been proposed as risk factors for both DM and CVD [3, 7, 8, 17–23]. Among them, several markers have already been widely studied, such as total and differential leukocyte counts [17–23] and C-reactive protein (CRP) [3, 7, 8]. However, correlations among markers have usually been weak or moderate [24, 25], which indicates that they potentially reflect different aspects of inflammation. For clinical purposes, it is necessary to identify reliable and specific markers that can help to discriminate between elevated risks of DM and CVD to enable targeted therapies.

Therefore, we conducted the present study to investigate the inflammatory profiles of DM and CVD and to distinguish their shared and specific markers.

## Materials and methods

### Participants

The Malmö Diet and Cancer study (MDCS) is a large prospective cohort study. Subjects were recruited from the general population from Malmö, a southern city of Sweden (participation rate: 40.8%) [26]. A baseline examination was conducted on 11,246 males and 17,203 females between March 1991 and September 1996 and included peripheral venous blood samples, physical examination, and a self-administered questionnaire. Of the initial 28,449 participants, complete information on leukocyte counts and covariates was available for 27,952 participants (Additional file 1). Twenty-two subjects with total leukocyte count higher than 20 × 10^9^/L were excluded to rule out severe inflammation [27]. We further excluded 1214 subjects with previous DM and 747 subjects with previous CVD. The final study population consisted of 25,969 participants (9843 males and 16,126 females, aged 45–73 years). Based on this cohort, total and differential leukocyte counts, as well as neutrophil to lymphocyte ratio (NLR), were studied in relation to DM and CVD (cohort analysis 1).

In addition, another seven inflammatory markers, including ceruloplasmin, alpha1-antitrypsin, orosomucoid, haptoglobin, complement C3, CRP and soluble urokinase plasminogen activator receptor (suPAR), were investigated in relation to DM and CVD using a sub-cohort of MDCS, the Malmö Diet and Cancer Cardiovascular cohort study (MDC–CV) [28]. In the MDC–CV, 6103 participants were randomly selected from the MDCS between 1991 and 1994 to study the epidemiology of carotid artery disease. From the MDC–CV 875 subjects were excluded due to missing data on waist circumference, smoking, low-density lipoprotein (LDL), or fasting glucose. Four hundred and 62 subjects with DM [i.e., self-reported DM, use of diabetes medication, fasting whole blood glucose of ≥ 6.1 mmol/L (corresponding to a fasting plasma glucose of ≥ 7.0 mmol/L [29]) or DM according to national or local patient registers] and 108 subjects with CVD were also excluded at baseline. Finally, we excluded those with missing information for each analysis of the specific inflammatory marker. Therefore, different cohort analyses (cohort analyses 2–8) were conducted on slightly different populations when investigating different markers (Additional file 1).

In sub-analyses (sub-analyses 1–8), those who developed both DM and CVD during follow-up, regardless of order, were additionally excluded in each cohort analysis. The study population flow chart is illustrated in Additional file 1.

All procedures performed in this study were in accordance with the ethical standards of the regional ethics committee in Lund, Sweden (LU 51/90) and with the 1964 Helsinki declaration and its later amendments or comparable ethical standard. Informed consent was obtained from all individual participants included in the study.

### Baseline examinations

After 10 min of rest in the supine position, blood pressure was measured using a mercury-column sphygmomanometer. Waist circumference was measured in the midpoint between the iliac crest and the lowest rib. Data on smoking habits, use of antihypertensive or antidiabetic medications were derived from a self-administered questionnaire. Cigarette smoking status was categorized as current smokers and non-smokers.

### Laboratory measurements

Fasting blood samples were drawn from the cubital vein. Blood glucose and blood cell counts were measured on the same day of blood sampling, according to standard procedures at the Department of Clinical Chemistry, University Hospital Malmö. Total leukocyte count and counts of leukocyte subtypes, including neutrophils, lymphocytes and a group of mixed cell types (monocytes, eosinophils and basophils), were measured in heparinized blood samples using a SYSMEX K1000 automated hematology analyzer (Sysmex Europe, Norderstedt, Germany). The coefficient of variations (CVs) for 20 consecutive counts of total leukocytes, neutrophils, lymphocytes, and mixed cells in one blood sample were 1.45, 1.85, 6.22, and 16.1%, respectively. The measurement range was 1.00 × 10^9^–99.9 × 10^9^/L for total leukocyte count. NLR was calculated as the ratio of neutrophil and lymphocyte counts that were measured in the same blood sample. The LDL concentration was calculated according to the Friedewald’s formula.

Fasting plasma samples for measuring other biomarkers were stored at − 80 °C immediately after collection. Ceruloplasmin, alpha1-antitrypsin, orosomucoid, haptoglobin and C3 were measured by Cobas c-systems (Roche Diagnostics GmbH, Germany) [30]. The measurement range was between 0.03 and 1.40 g/L for ceruloplasmin, 0.20 and 6.00 g/L for alpha1-antitrypsin, 0.1 and 4.0 g/L for orosomucoid, 0.10 and 5.70 g/L for haptoglobin, and 0.04 and 5.00 g/L for C3, respectively. The CVs were < 5%. CRP was measured by the Tina-quant^®^ CRP latex high sensitivity assay (Roche Diagnostics, Basel, Switzerland) on an ADVIA 1650 Chemistry System (Bayer Healthcare, NY, USA). Study samples were analysed as discrete samples and results were read in 6 s intervals for a 1 min time period following 5 min of incubation. The reported result was the mean value of these measurements. SuPAR was measured by an enzyme-linked immunosorbent assay suPARnostic^®^ kit (ViroGates, Copenhagen, Denmark), with a validated range between 0.6 and 22.0 ng/mL [31]. The inter- and intra-assay CVs were 9.17 and 2.75%, respectively [31].

### Ascertainment of endpoints

Participants were followed from baseline examination until death, migration from Sweden, end of follow-up (December 31st, 2014), or first diagnosis of DM/CVD, whichever came first.

Information on vital status and emigration was provided by the Swedish population register, and information on incident DM [32] and CVD [33–35] was retrieved from local and national registers that have previously been described and validated [32–35]. In short, new cases of DM were identified in the Malmö HbA_1c_ register (MHR), the Swedish National Diabetes Register (NDR), the regional Diabetes 2000 register of the Scania region, the Swedish inpatient register, the Swedish outpatient register, and the nationwide Swedish drug prescription register. At least two independent sources confirmed the diagnosis for 74% of the cases. Since 1988, the MHR at the Department of Clinical Chemistry, Malmö University Hospital, analyzed and recorded HbA_1c_ samples taken in institutional and non-institutional care in the greater Malmö area. In the MHR, individuals were considered to have incident DM if they had at least two HbA_1c_ recordings ≥ 6.0% (42 mmol/mol) with the Swedish Mono-S standardization system [corresponding to 7.0% (53 mmol/mol) according to the US National Glycohemoglobin Standardization Program] during follow-up. In the NDR and the Diabetes 2000 register, DM was diagnosed using established diagnostic criteria (fasting glucose concentration in plasma ≥ 7.0 mmol/L with repeated tests). In the Swedish inpatient register and outpatient register, DM was diagnosed by a senior physician, while in the nationwide prescription register, a filled prescription of insulin or antidiabetic medications (ATC-code A10) was required for diagnosing DM. For CVD (i.e., coronary event or stroke), the national Swedish Hospital Discharge register, the Cause-of-Death register [33, 34] and the Stroke register of Malmö [35] were used to identify new cases of coronary event (fatal or nonfatal myocardial infarction) or stroke throughout the follow-up period. A coronary event was defined based on the International Classification of Diseases 9th (ICD-9) codes 410A-410X and ICD-10 code I21 or death attributable to ischemic heart disease (ICD-9 codes: 410–414; ICD-10 codes: I20–I25). Stroke was defined as ICD-9 codes 430, 431,434 or 436, or ICD-10 codes I60, I61 or I63–64.

### Statistical analyses

Participants were categorized into four groups: (1) participants who developed neither DM nor CVD during follow-up; (2) participants who developed DM but not CVD during follow-up; (3) participants who developed CVD but not DM during follow-up; (4) participants who developed both DM and CVD during follow-up. Because the distribution of data on CRP was skewed, a natural logarithm transformation was applied to normalize the data. The age- and sex-adjusted characteristics of the participants are presented as geometric means (95% confidence intervals, CIs) for continuous variables, and percentages for categorical variables. Analysis of covariance was used for continuous variables and multiple logistic regression analysis was used for categorical variables, to compare differences in baseline characteristics across the four groups as well as between groups 2 and 3, after adjusting for age and sex. Pearsons’ correlation test was performed to examine the relation between each two inflammatory markers. Incident DM and incident CVD were studied in relation to total leukocyte count, neutrophil count, lymphocyte count, mixed cell count and NLR (one biomarker at a time), based on the MDCS cohort (cohort analysis 1). Meanwhile, based on MDC–CV, the two outcomes were also studied in relation to ceruloplasmin, alpha1-antitrypsin, orosomucoid, haptoglobin, C3, CRP, and suPAR (one biomarker at a time), in another seven sets of analyses (cohort analyses 2–8). For each set of analyses, two separate analyses were conducted using either incident DM or incident CVD as the outcome. Cox regression with time-on-study as time-scale was used to estimate hazard ratios (HRs) and 95% CIs for incident DM or CVD per one standard deviation increase in each inflammatory marker. Potential confounders in cohort analysis 1 included age, sex, waist circumference, smoking, systolic blood pressure, and use of anti-hypertensive medications. Cohort analyses 2–8 additionally included LDL-cholesterol.

We were interested in investigating whether the risk associated with each inflammatory marker was similar for the separate endpoints of DM and CVD. For this purpose, we used a competing risks approach described by Lunn and McNeil [36]. Briefly, the dataset was duplicated with two rows per individual, and was stratified on these rows. The endpoints of DM and CVD were separated into these strata. Unlike the original method [36], in this study individuals had events in both strata if they developed both DM and CVD during follow-up. An analysis was then conducted with duplicated covariates so that each covariate was allowed to have different effects in each stratum. The HRs (95% CIs) derived from this analysis are identical to those obtained from separate Cox models fitted in the original dataset. Another analysis is then conducted, with one variable un-duplicated. This forces the effect measure for this variable to be the same for both strata. This analysis is then compared to the one with differential effects using the likelihood ratio test, with one degree of freedom, in order to derive a *p*-value for the difference in effect measures for the covariate. These steps were repeated for each inflammatory marker. Since all the subjects included in cohort analyses 2–8 were eligible for the diagnosis of the metabolic syndrome (MetS), cohort analyses 2–8 and the corresponding comparisons between HRs for DM versus CVD were conducted again, adjusted for age, sex, smoking, and the MetS (defined according to the NCEP-ATPIII criteria [37, 38]). Sensitivity analyses were also conducted using coronary event and stroke as separate outcomes. Furthermore, both the age and sex-adjusted analyses and multivariate analyses were repeated after excluding those who developed both DM and CVD during follow-up (group 4).

All analyses were performed using the Statistical Analysis System version 9.3 for Windows (SAS Institute Inc., Cary, NC, USA).

## Results

### Baseline characteristics

In MDCS (n = 25,969), there were 3819 DM events (mean follow-up of 17.4 ± 5.58 years) and 4548 CVD events (mean follow-up of 17.7 ± 5.46 years). 982 individuals had both DM and CVD events. In MDC–CV (n = 4658), there were 646 DM events (mean follow-up of 19.0 ± 5.16 years) and 783 CVD events (mean follow-up of 19.0 ± 5.35 years). Among them, 151 individuals had both DM and CVD. The incidence rates of DM and CVD were 8.42 and 9.90 per 1000 person-years, respectively, in MDCS, and 7.30 and 8.83 per 1000 person-years respectively, in MDC–CV.

Baseline characteristics of participants according to DM and CVD event status during follow-up are presented in Table 1. Compared with those who developed only DM (group 2), individuals with only CVD (group 3) tended to be older, have lower waist circumstance, lower systolic blood pressure or less use of anti-hypertensive medications. Moreover, a larger proportion of these subjects were males or smokers. In terms of inflammatory markers, levels of lymphocyte count, C3 and CRP were lower, while levels of neutrophil count, NLR and alpha1-antitrypsin were higher in group 3, when compared with group 2.

**Table 1:** Baseline characteristics according to incidence of diabetes (DM) and cardiovascular disease (CVD) during follow-up

In MDCS (n = 25,969)	Group1 without DM or CVD	Group2 incident DM alone	Group3 incident CVD alone	Group4 incident DM and CVD	*p* for trend^a^
	n = 18,584	n = 2837	n = 3566	n = 982	
Age (years)	57.3 (57.2, 57.4)^b^	57.4 (57.1, 57.7)^c^	61.8 (61.6, 62.1)^c^	60.4 (59.9, 60.9)	< 0.001
Sex (male, %)	33.1	44.3^c^	52.7^c^	56.4	< 0.001
Waist circumference (cm)	84.0 (83.9, 84.2)	91.6 (91.2, 91.9)^c^	85.2 (84.8, 85.5)^c^	91.8 (91.2, 92.4)	< 0.001
Systolic blood pressure (mmHg)	139.2 (138.9, 139.5)	145.2 (144.5, 145.8)^c^	143.9 (143.3, 144.5)^c^	149.5 (148.4, 150.7)	< 0.001
Smokers (%)	27.4	27.9^c^	33.0^c^	34.2	< 0.001
Anti-hypertensive medication (%)	12.4	24.2^c^	21.2^c^	31.2	< 0.001
Total leukocyte count (10^9^/L)	6.23 (6.21, 6.26)	6.59 (6.53, 6.66)	6.58 (6.52, 6.63)	6.82 (6.72, 6.92)	< 0.001
Neutrophil count (10^9^/L)	3.82 (3.80, 3.84)	4.01 (3.96, 4.06)^c^	4.07 (4.02, 4.11)^c^	4.21 (4.13, 4.29)	< 0.001
Lymphocyte count (10^9^/L)	1.90 (1.89, 1.91)	2.04 (2.02, 2.06)^c^	1.98 (1.95, 2.00)^c^	2.05 (2.01, 2.09)	< 0.001
Mixed cell count (10^9^/L)	0.517 (0.514, 0.520)	0.541 (0.534, 0.549)	0.537 (0.530, 0.543)	0.558 (0.545, 0.571)	< 0.001
NLR	2.15 (2.14, 2.17)	2.10 (2.07, 2.14)^c^	2.20 (2.17, 2.23)^c^	2.18 (2.13, 2.24)	0.004

In MDC–CV (n = 4658)	n = 3380	n = 495	n = 632	n = 151	
Age (years)	56.9 (56.6, 57.1)	56.7 (56.2, 57.2)	60.1 (59.6, 60.5)	59.3 (58.4, 60.2)	< 0.001
Sex (male, %)	35.7	38.0^c^	52.1^c^	54.3	< 0.001
Waist circumference (cm)	83.3 (83.0, 83.6)	88.7 (87.9, 89.5)	84.4 (83.7, 85.2)	90.2 (88.7, 91.6)	0.007
Systolic blood pressure (mmHg)	139.2 (138.6, 139.8)	144.3 (142.7, 145.8)	144.2 (142.9, 145.6)	150.3 (147.5, 153.1)	< 0.001
Low-density lipoprotein cholesterol (mmol/L)	4.12 (4.09, 4.16)	4.30 (4.21, 4.38)	4.19 (4.11, 4.27)	4.33 (4.17, 4.48)	0.112
Smokers (%)	21.5	22.2^c^	29.1^c^	27.8	< 0.001
Anti-hypertensive medication (%)	11.0	21.2^c^	18.7^c^	25.2	< 0.001
Ceruloplasmin (g/L) (missing = 536)	0.500 (0.496, 0.505)	0.503 (0.492, 0.514)	0.513 (0.503, 0.523)	0.516 (0.496, 0.537)	0.020
Alpha1-antitrypsin (g/L) (missing = 359)	1.20 (1.19, 1.21)	1.20 (1.17, 1.22)^c^	1.24 (1.22, 1.26)^c^	1.24 (1.19, 1.29)	0.002
Orosomucoid (g/L) (missing = 336)	0.693 (0.685, 0.700)	0.752 (0.732, 0.771)	0.731 (0.714, 0.748)	0.763 (0.728, 0.797)	< 0.001
Haptoglobin (g/L) (missing = 667)	1.28 (1.26, 1.30)	1.36 (1.31, 1.42)	1.34 (1.29, 1.39)	1.46 (1.36, 1.55)	0.020
C3 (g/L) (missing = 289)	1.50 (1.49, 1.51)	1.66 (1.63, 1.69)^c^	1.54 (1.52, 1.57)^c^	1.68 (1.62, 1.73)	0.004
CRP (mg/L) (missing = 187)	2.25 (2.1, 2.39)	3.09 (2.71, 3.47)^c^	2.66 (2.31, 3.00)^c^	3.75 (3.05, 4.46)	0.030
SuPAR (ng/mL) (missing = 149)	2.93 (2.9, 2.96)	3.04 (2.96, 3.12)	3.16 (3.08, 3.23)	3.07 (2.92, 3.22)	< 0.001

*NLR* neutrophil to lymphocyte ratio, *CRP* C-reactive protein, *SuPAR* soluble urokinase plasminogen activator receptor

^a^Analysis of covariance or multiple logistic regression analysis, adjusted for age and sex

^b^Geometric mean (95% confidence interval) (all such values)

^c^Significant difference was found when participants with incident DM alone were compared with those with CVD alone

Correlations between each two inflammatory markers are reported in Table 2. Moderate correlations were observed among most of the markers.

**Table 2:** Pearson correlations between every two inflammatory markers

In MDCC (n = 25,969)	Total leukocyte count	Neutrophil count	Lymphocyte count	Mixed cell count	NLR		
Neutrophil count	0.91	–	–	–	–		
Lymphocyte count	0.61	0.25	–	–	–		
Mixed cell count	0.45	0.27	0.30	–	–		
NLR	0.31	0.62	-0.48	–	–		

In MDCC (n = 3630)	Ceruloplasmin	Alpha1-antitrypsin	Orosomucoid	Haptoglobin	C3	CRP	SuPAR
Alpha1-antitrypsin	0.52	–	–	–	–	–	–
Orosomucoid	0.45	0.40	–	–	–	–	–
Haptoglobin	0.43	0.41	0.60	–	–	–	–
Complement C3	0.52	0.41	0.60	0.51	–	–	–
CRP	0.38	0.27	0.52	0.44	0.40	–	–
SuPAR	0.13	0.17	0.22	0.19	0.08	0.23	–
Total leukocyte count	0.07	0.12	0.17	0.20	0.09	0.20	0.21
Neutrophil count	0.05	0.12	0.16	0.17	0.06	0.16	0.18
Lymphocyte count	0.06	–	0.07	0.14	0.11	0.14	0.14
Mixed cell count	–	0.06	0.12	0.12	0.11	0.14	0.13
NLR	–	0.10	0.10	0.07	–	0.06	0.09

*NLR* neutrophil to lymphocyte ratio, *CRP* C-reactive protein, *SuPAR* soluble urokinase plasminogen activator receptor

*r* > 0.05 is statistically significant, *p *< 0.01

### Inflammatory markers for prediction of incident DM and CVD

The age- and sex-adjusted HRs of biomarkers for incident DM and CVD are summarized in Table 3, and the multivariate-adjusted HRs are summarized in Table 4. After adjustment for conventional risk factors, total leukocyte count, neutrophil count, lymphocyte count, mixed cell count, orosomucoid and CRP were associated with an increased risk of both DM and CVD. In addition, NLR, ceruloplasmin, alpha1-antitrypsin, and SuPAR predicted increased risk of CVD but not DM, while haptoglobin and C3 predicted increased risk of DM but not CVD.

**Table 3:** Comparison of risk of diabetes and cardiovascular disease in relation to inflammatory markers (age, sex-adjusted)

Inflammatory markers	No. of subjects	Diabetes			Cardiovascular disease			*p* value for equal associations^c^
		Incidence	HR (95% CI)^a^	*p* ^b^	Incidence	HR (95% CI)^a^	*p* ^b^	
In MDCS								
Total leukocyte count	25,969	3819	1.249 (1.214, 1.285)	< 0.001	4548	1.235 (1.202, 1.269)	< 0.001	0.572
Neutrophil count	25,969	3819	1.182 (1.148, 1.216)	< 0.001	4548	1.209 (1.177, 1.242)	< 0.001	0.245
Lymphocyte count	25,969	3819	1.171 (1.147, 1.196)	< 0.001	4548	1.105 (1.081, 1.130)	< 0.001	< 0.001
Mixed cell count	25,969	3819	1.150 (1.116, 1.185)	< 0.001	4548	1.109 (1.079, 1.140)	< 0.001	0.080
NLR	25,969	3819	0.981 (0.949, 1.014)	0.265	4548	1.058 (1.030, 1.088)	< 0.001	< 0.001
In MDC–CV								
Ceruloplasmin	4122	543	1.056 (0.967, 1.153)	0.225	678	1.123 (1.040, 1.213)	0.003	0.300
Alpha1-antitrypsin	4299	583	1.042 (0.959, 1.133)	0.330	709	1.167 (1.086, 1.254)	< 0.001	0.044
Orosomucoid	4322	591	1.290 (1.203, 1.384)	< 0.001	716	1.185 (1.109, 1.267)	< 0.001	0.087
Haptoglobin	3991	520	1.235 (1.138, 1.339)	< 0.001	651	1.171 (1.087, 1.262)	< 0.001	0.351
C3	4369	598	1.452 (1.362, 1.547)	< 0.001	722	1.151 (1.075, 1.232)	< 0.001	< 0.001
CRP	4471	615	1.413 (1.306, 1.529)	< 0.001	729	1.244 (1.157, 1.337)	< 0.001	0.020
SuPAR	4509	618	1.149 (1.068, 1.237)	< 0.001	751	1.261 (1.184, 1.342)	< 0.001	0.060

*HR* hazard ratio, *CI* confidence interval, *NLR* neutrophil to lymphocyte ratio, *CRP* C-reactive protein, *SuPAR* soluble urokinase plasminogen activator receptor

^a^Age- and sex-adjusted hazard ratios and 95% confidence intervals, per 1 standard deviation (all such values)

^b^Analysis by Cox proportional hazards model

^c^*p* value associated with the null hypothesis that this variable has the same association with diabetes and cardiovascular disease, with all other effects being different; tests for all variables have 1 df

**Table 4:** Comparison of risk of diabetes and cardiovascular disease in relation to inflammatory markers (multivariate analysis)

Inflammatory markers	No. of subjects	Diabetes			Cardiovascular disease			*p* value for equal associations^c^
		Incidence	HR (95% CI)^a^	*p* ^b^	Incidence	HR (95% CI)^a^	*p* ^b^	
In MDCS^d^								
Total leukocyte count	25,969	3819	1.118 (1.082, 1.154)	< 0.001	4548	1.114 (1.081, 1.148)	< 0.001	0.888
Neutrophil count	25,969	3819	1.086 (1.052, 1.121)	< 0.001	4548	1.108 (1.076, 1.140)	< 0.001	0.365
Lymphocyte count	25,969	3819	1.092 (1.060, 1.124)	< 0.001	4548	1.040 (1.012, 1.070)	< 0.001	0.020
Mixed cell count	25,969	3819	1.054 (1.021, 1.087)	< 0.001	4548	1.050 (1.020, 1.080)	< 0.001	0.862
NLR	25,969	3819	0.998 (0.966, 1.032)	0.929	4548	1.049 (1.020, 1.078)	< 0.001	0.025
In MDC–CV^e^								
Ceruloplasmin	4122	543	1.002 (0.912, 1.100)	0.971	678	1.087 (1.002, 1.179)	0.045	0.197
Alpha1-antitrypsin	4299	583	1.025 (0.942, 1.116)	0.565	709	1.113 (1.033, 1.199)	0.005	0.154
Orosomucoid	4322	591	1.118 (1.032, 1.211)	0.006	716	1.088 (1.011, 1.171)	0.024	0.626
Haptoglobin	3991	520	1.110 (1.017, 1.210)	0.019	651	1.061 (0.979, 1.149)	0.149	0.455
C3	4369	598	1.250 (1.160, 1.347)	< 0.001	722	1.076 (0.997, 1.161)	0.059	0.006
CRP	4471	615	1.174 (1.076, 1.280)	< 0.001	729	1.116 (1.033, 1.206)	0.005	0.396
SuPAR	4509	618	1.041 (0.956, 1.133)	0.360	751	1.137 (1.059, 1.221)	< 0.001	0.117

*HR* hazard ratio, *CI* confidence interval, *NLR* neutrophil to lymphocyte ratio, *CRP* C-reactive protein, *SuPAR* soluble urokinase plasminogen activator receptor

^a^Adjusted hazard ratios and 95% confidence intervals, per 1 standard deviation (all such values)

^b^Analysis by Cox proportional hazards model

^c^*p* value associated with the null hypothesis that this variable has the same association with diabetes and cardiovascular disease, with all other effects being different; tests for all variables have 1 df

^d^Adjusted for age, sex, waist circumference, smoking, systolic blood pressure, and anti-hypertensive drug medication

^e^Adjusted for age, sex, waist circumference, smoking, systolic blood pressure, low-density lipoprotein, and anti-hypertensive drug medication

Inflammatory profiles for DM and CVD were generally consistent in secondary analyses excluding those who developed both DM and CVD (Additional files 2, 3). However, the association of haptoglobin with DM that was initially observed in Table 4 was statistically nonsignificant in this sub-group (Additional file 3, fully adjusted model).

### Comparisons of inflammatory markers for incident DM and CVD

The analyses of differential effects between DM and CVD outcomes for each specific inflammatory marker are presented in Tables 3 and 4. After adjusting for age and sex (Table 3), several markers showed differential associations with DM versus CVD. After adjusting for other potential confounders (Table 4), only the associations of lymphocyte count, NLR and C3 showed significantly different effects for DM versus CVD (*p* value for equal associations = 0.020, = 0.025 and = 0.006, respectively). Both lymphocyte count and C3 had stronger associations with risk of DM compared with CVD, and the predictive value of C3 for DM was considerably stronger than for CVD. By contrast, NLR was associated with an increased risk of CVD but was not associated with DM. In sensitivity analyses, results were substantially unchanged when age, sex, smoking, and the MetS were adjusted for in multivariate analyses (data not shown). Likewise, consistent results were observed after excluding those who developed both DM and CVD during follow-up (Additional file 3, fully adjusted model). The additional analyses using coronary event and stroke as separate outcomes revealed that the inequal associations of NLR with DM versus CVD were mainly contributed by coronary events, while the inequal associations of lymphocyte count and C3 with DM versus CVD were mainly contributed by stroke events (data not shown).

## Discussion

To our knowledge, this is the first cohort study comparing the predictive values of different inflammatory markers for the risk of DM versus CVD. Overall, the results indicated substantial similarities in the inflammatory profiles associated with DM and CVD. Lymphocyte count, NLR and C3 were associated differently with DM versus CVD. In particular, C3 showed a remarkable difference in its association with DM compared to CVD. Taken together, the results suggest that there is an inflammatory ‘common soil’ for DM and CVD, but there are also important differences.

Previous genetic studies have supported an overlap between the pathogenesis of DM and CVD [5, 6]. Traditional CVD risk factors, including inflammation and endothelial dysfunction, have also been found to be positively associated with incident DM [2, 3]. The MetS predisposes to both DM and CVD, which are considered to be particularly triggered by IR and atherosclerosis, respectively [6]. These two disorders have been shown by gene set enrichment analysis to have shared dysregulated pathways, many of which are involved in inflammatory responses [6]. Nevertheless, it is noteworthy that the MetS was differently associated with DM versus CVD [9–11]. In addition, acute-phase proteins exhibit different predictive ability for changes in insulin secretion, insulin sensitivity, and cardiometabolic traits, indicating that different inflammatory cytokines capture distinct aspects of inflammation [15]. In the recently reported Canakinumab Anti-inflammatory Thrombosis Outcome Study (CANTOS), canakinumab, an inflammation inhibitor targeting at interleukin-1ß, showed significant effect on cardiovascular events [14]. However, despite marked reductions in CRP and interleukin-1, it did not reduce incident DM [12], suggesting divergence of inflammatory pathways in CVD and DM. Therefore, specific anti-inflammatory targets for therapeutic interventions need to be recognized for individuals with DM and CVD, respectively [13].

The relationship between C3 and risk of DM in the general population supports the conclusion from other prospective studies [24, 29, 39, 40] demonstrating that elevated C3 levels are associated with an increased risk of IR, prediabetes and DM. In addition, elevated C3 concentration was shown to be a stronger predictor of DM than multiple other acute-phase proteins [24, 29]. The C3-dependent immune response may contribute to destruction of pancreatic islets and hepatic dysfunction [41, 42], both of which are important risk factors for hyperglycemia [7, 43]. Another possible pathophysiological link is that the proteolytic fragments of C3, C3a and C5a, may mediate adipose tissue inflammation and IR [44, 45], and thus facilitate DM development [7]. Prospective studies of C3 and incident CVD are relatively few, especially in apparently healthy subjects [46, 47]. Muscari et al. have demonstrated an increased risk of CVD in men with high C3 levels, but these analyses were only age-adjusted [46]. Engström et al. have confirmed this finding, but also noted that the observed association was completely attenuated and non-significant after adjusting for potential confounders including baseline DM [47]. In line with these studies, we observed a C3-CVD association in models adjusted for age and sex, but not in fully adjusted models. It has been suggested that, in DM, the interaction between C3 and fibrinogen may be responsible for impaired fibrinolysis and subsequent CVD risk [48].

Total and differential leukocyte counts are traditional markers of subclinical inflammation and their associations with risk of DM and CVD have been widely reported in epidemiological studies [17–23]. In accordance with previous findings, we demonstrated that, among the major leukocyte subtypes, the strongest risk prediction for CVD was given by neutrophil count [17, 18, 20–22], and the strongest risk prediction for DM was given by lymphocyte count [19, 23]. Neutrophil infiltration has been observed both in atherosclerosis in the aorta of atherosclerotic mice [49] and in culprit lesions in patients with acute coronary syndromes [50]. Neutrophils may mediate cardiovascular anomalies by interacting with endothelium and platelets [49, 51], leading to plaque destabilization [52]. As for DM, the predictive ability of lymphocyte count was the strongest, but still rather similar to that of neutrophil count [23]. Both neutrophils and lymphocytes have been found to be critical to obesity-associated adipose tissue inflammation, which subsequently contributes to IR and DM [53–55]. In addition to previous evidence, we further demonstrated that neutrophil count was similarly associated with DM and with CVD while the predictive ability of lymphocyte count for DM was superior to that for CVD. NLR shows the combined effect of neutrophil count and lymphocyte count. Whereas its prognostic value in CVD has been widely discussed [18, 56], controversy has remained regarding whether NLR is an efficient predictor for DM [19, 57]. In the present study, NLR was found to be predictive for CVD but not DM. Similarly, NLR was not associated with IR or adiposity in previous studies [19, 58, 59]. It is noteworthy that despite a strong association of lymphocyte count with DM, the role of lymphocyte count in CVD seems to be weak or even inverse [17, 18, 20–22]. The association of NLR with CVD may be driven by neutrophil-related processes, rather than by adiposity or IR.

This study used a large sample with long-term follow-up. Outcome data were retrieved from validated hospital registers with national coverage [32–35], which should reduce information bias. The competing risks analyses were conducted before and after excluding those with both incident DM and incident CVD, which helps to distinguish between the shared and specific markers for DM and CVD. Our study is limited by lack of information on other potential inflammatory markers. In addition, DM types were not distinguished. However, all participants were middle-aged adults. Because people with type 1 DM usually show symptoms in early life, they should have been thoroughly excluded at baseline as prevalent cases. Therefore, all cases of incident DM in this study were very likely to be type 2 DM.

## Conclusions

There are many similarities, but also important differences in the associations of inflammatory markers with DM versus CVD. Most importantly, C3 was an efficient and highly specific predictor of DM. In addition, lymphocyte count showed superior predictive value for DM than for CVD, while NLR was a specific and moderate predictor of CVD, but not for DM. Our results may help discriminate between individuals at elevated risk of DM and those at elevated risk of CVD, which is a prerequisite for targeted therapies.

## Additional files

**Additional file 1.** Study population flow chart. **Figure S1.** *MDCS* the Malmö Diet and Cancer study, *MDC-CV* the Malmö Diet and Cancer Cardiovascular cohort study, *CVD* cardiovascular disease, *LDL* low-density lipoprotein, *CRP* C-reactive protein, *SuPAR* soluble urokinase plasminogen activator receptor.

**Additional file 2.** Comparison of risk of diabetes and cardiovascular disease in relation to inflammatory markers (age, sex-adjusted analysis excluding those who developed both diabetes and cardiovascular disease).

**Additional file 3.** Comparison of risk of diabetes and cardiovascular disease in relation to inflammatory markers (multivariate analysis excluding those who developed both diabetes and cardiovascular disease).

## Abbreviations

CANTOS: Canakinumab Anti-inflammatory Thrombosis Outcome Study
CI: confidence interval
CRP: C-reactive protein
CV: coefficient of variation
CVD: cardiovascular disease
DM: diabetes mellitus
HR: hazard ratio
IR: insulin resistance
ICD: International Classification of Diseases
LDL: low-density lipoprotein
MDCS: Malmö Diet and Cancer study
MDC–CV: Malmö Diet and Cancer Cardiovascular cohort study
MetS: metabolic syndrome
MHR: Malmö HbA1c register
NDR: National Diabetes Register
NLR: neutrophil to lymphocyte ratio
SuPAR: soluble urokinase plasminogen activator receptor
