# Supplementary material for: Comparing the inflammatory profiles for incidence of diabetes mellitus and cardiovascular diseases: a prospective study exploring the ‘common soil’ hypothesis
Source: Cardiovasc Diabetol. 2018 Jun 12;17:87. doi: 10.1186/s12933-018-0733-9 (PMC5996509; doi:10.1186/s12933-018-0733-9)
Supplement: Supplementary file 1 — Additional file 1. Study population flow chart. Figure S1. MDCS the Malmö Diet and Cancer study, MDC-CV the Malmö Diet and Cancer Cardiovascular cohort study, CVD cardiovascular disease, LDL low-density lipoprotein, CRP C-reactive protein, SuPAR soluble urokinase plasminogen activator receptor. [file 12933_2018_733_MOESM1_ESM.docx]

ceruloplasmin

alpha1-antitrypsin

orosomucoid

haptoglobin

complement

C3

CRP

suPAR

Total and differential leukocyte counts

MDCS cohort, n=28449

Missing covariables=497

(Waist circumference=61

Smoking=322

Blood pressure=30

Leukocyte counts=84)

Baseline CVD=747

Total leukocyte count

>20×10^9^/L=22

Cohort analysis 1. n=25969, aged 45-73 years, 37.9% men

Baseline diabetes=1214

Sub-analysis 1: cases excluded with both diabetes and CVD during follow-up=982

MDC-CV cohort, n=6103

Missing covariables=875

(Waist circumference=10

Smoking=281

LDL=565

Glucose=19)

Baseline diabetes=462

Baseline CVD=108

cases excluded with both incident diabetes and CVD

cases excluded with both incident diabetes and CVD

Analysis 2.

n=4,122

n=536

Sub-analysis 2

Missing value

n=126

Analysis 3.

n=4,299

n=359

Sub-analysis 3

n=136

Analysis 4.

n=4,322

n=336

Sub-analysis 4

n=139

Analysis 5.

n=3,991

n=667

Sub-analysis 5

n=123

Analysis 6.

n=4,369

n=289

Sub-analysis 6

n=140

Missing value

Analysis 7.

n=4,471

n=187

Sub-analysis 7

n=139

Analysis 8.

n=4,509

n=144

Sub-analysis 8

n=144

Extreme value n=5

n=4658
