# Supplementary material for: Comparing the inflammatory profiles for incidence of diabetes mellitus and cardiovascular diseases: a prospective study exploring the ‘common soil’ hypothesis
Source: Cardiovasc Diabetol. 2018 Jun 12;17:87. doi: 10.1186/s12933-018-0733-9 (PMC5996509; doi:10.1186/s12933-018-0733-9)
Supplement: Supplementary file 2 — Additional file 2. Comparison of risk of diabetes and cardiovascular disease in relation to inflammatory markers (age, sex-adjusted analysis excluding those who developed both diabetes and cardiovascular disease). [file 12933_2018_733_MOESM2_ESM.docx]

| **Additional file 2** Comparison of risk of diabetes and cardiovascular disease in relation to inflammatory markers (age, sex-adjusted analysis excluding those who developed both diabetes and cardiovascular disease) | | | | | | | | | | | |
| --- | --- | --- | --- | --- | --- | --- | --- | --- | --- | --- | --- |
| Inflammatory markers | No. of subjects |  | Diabetes | | |  | Cardiovascular disease | | |  | *p* value for equal associations ^c^ |
|  |  |  | Incidence | HR (95% CI) ^a^ | *p* ^b^ |  | Incidence | HR (95% CI) ^a^ | *p* ^b^ |  |  |
| In MDCS |  |  |  |  |  |  |  |  |  |  |  |
| Total leukocyte count | 24987 |  | 2837 | 1.216 (1.170, 1.264) | <0.001 |  | 3566 | 1.210 (1.174, 1.247) | <0.001 |  | 0.841 |
| Neutrophil count | 24987 |  | 2837 | 1.150 (1.112, 1.190) | <0.001 |  | 3566 | 1.189 (1.153, 1.227) | <0.001 |  | 0.151 |
| Lymphocyte count | 24987 |  | 2837 | 1.176 (1.147, 1.205) | <0.001 |  | 3566 | 1.089 (1.060, 1.118) | <0.001 |  | <0.001 |
| Mixed cell count | 24987 |  | 2837 | 1.134 (1.095, 1.174) | <0.001 |  | 3566 | 1.089 (1.056, 1.124) | <0.001 |  | 0.095 |
| NLR | 24987 |  | 2837 | 0.959 (0.922, 0.998) | 0.040 |  | 3566 | 1.063 (1.030, 1.096) | <0.001 |  | <0.001 |
|  |  |  |  |  |  |  |  |  |  |  |  |
| In MDC-CV |  |  |  |  |  |  |  |  |  |  |  |
| Ceruloplasmin | 3996 |  | 417 | 1.029 (0.930, 1.138) | 0.585 |  | 552 | 1.128 (1.036, 1.229) | 0.006 |  | 0.171 |
| Alpha1-antitrypsin | 4163 |  | 447 | 1.004 (0.913, 1.104) | 0.937 |  | 573 | 1.171 (1.082, 1.268) | <0.001 |  | 0.015 |
| Orosomucoid | 4183 |  | 452 | 1.281 (1.183, 1.388) | <0.001 |  | 577 | 1.166 (1.083, 1.256) | <0.001 |  | 0.091 |
| Haptoglobin | 3868 |  | 397 | 1.192 (1.085, 1.309) | <0.001 |  | 528 | 1.135 (1.043, 1.234) | 0.003 |  | 0.444 |
| C3 | 4229 |  | 458 | 1.441 (1.340, 1.549) | <0.001 |  | 582 | 1.088 (1.004, 1.177) | 0.038 |  | <0.001 |
| CRP | 4332 |  | 476 | 1.363 (1.246, 1.491) | <0.001 |  | 590 | 1.181 (1.090, 1.281) | <0.001 |  | 0.021 |
| SuPAR | 4365 |  | 474 | 1.095 (1.001, 1.198) | 0.046 |  | 607 | 1.275 (1.190, 1.366) | <0.001 |  | <0.001 |
| *HR* hazard ratio, *CI* confidence interval, *NLR* neutrophil lymphocyte ratio, *CRP* C-reactive protein, *SuPAR* soluble urokinase plasminogen activator receptor | | | | | | | | | | | |
| ^a^ Age- and sex-adjusted hazard ratios and 95% confidence intervals, per 1 standard deviation (all such values) | | | | | | | | | | | |
| ^b^ Analysis by Cox proportional hazards model | | | | | | | | | | | |
| ^c^ *p* value associated with the null hypothesis that this variable has the same association with diabetes and cardiovascular disease, with all other effects being different; tests for all variables have 1 df | | | | | | | | | | | |
