# Supplementary material for: Comparing the inflammatory profiles for incidence of diabetes mellitus and cardiovascular diseases: a prospective study exploring the ‘common soil’ hypothesis
Source: Cardiovasc Diabetol. 2018 Jun 12;17:87. doi: 10.1186/s12933-018-0733-9 (PMC5996509; doi:10.1186/s12933-018-0733-9)
Supplement: Supplementary file 3 — Additional file 3. Comparison of risk of diabetes and cardiovascular disease in relation to inflammatory markers (multivariate analysis excluding those who developed both diabetes and cardiovascular disease). [file 12933_2018_733_MOESM3_ESM.docx]

| **Additional file 3** Comparison of risk of diabetes and cardiovascular disease in relation to inflammatory markers (multivariate analysis excluding those who developed both diabetes and cardiovascular disease) | | | | | | | | | | | |
| --- | --- | --- | --- | --- | --- | --- | --- | --- | --- | --- | --- |
| Inflammatory markers | No. of subjects |  | Diabetes | | |  | Cardiovascular disease | | |  | *p* value for equal associations ^c^ |
|  |  |  | Incidence | HR (95% CI) ^a^ | *p* ^b^ |  | Incidence | HR (95% CI) ^a^ | *p* ^b^ |  |  |
| In MDCS ^d^ |  |  |  |  |  |  |  |  |  |  |  |
| Total leukocyte count | 24987 |  | 2837 | 1.102 (1.054, 1.153) | <0.001 |  | 3566 | 1.105 (1.069, 1.142) | <0.001 |  | 0.999 |
| Neutrophil count | 24987 |  | 2837 | 1.068 (1.029, 1.109) | <0.001 |  | 3566 | 1.098 (1.063, 1.135) | <0.001 |  | 0.273 |
| Lymphocyte count | 24987 |  | 2837 | 1.103 (1.067, 1.141) | <0.001 |  | 3566 | 1.038 (1.006, 1.071) | 0.020 |  | 0.010 |
| Mixed cell count | 24987 |  | 2837 | 1.044 (1.006, 1.083) | 0.021 |  | 3566 | 1.044 (1.011, 1.079) | 0.009 |  | 0.999 |
| NLR | 24987 |  | 2837 | 0.979 (0.941, 1.019) | 0.302 |  | 3566 | 1.046 (1.014, 1.080) | 0.005 |  | 0.010 |
|  |  |  |  |  |  |  |  |  |  |  |  |
| In MDC-CV ^e^ |  |  |  |  |  |  |  |  |  |  |  |
| Ceruloplasmin | 3996 |  | 417 | 0.980 (0.880, 1.091) | 0.708 |  | 552 | 1.101 (1.006, 1.204) | 0.036 |  | 0.102 |
| Alpha1-antitrypsin | 4163 |  | 447 | 0.998 (0.906, 1.100) | 0.971 |  | 573 | 1.120 (1.032, 1.216) | 0.007 |  | 0.075 |
| Orosomucoid | 4183 |  | 452 | 1.117 (1.021, 1.223) | 0.016 |  | 577 | 1.095 (1.010, 1.187) | 0.027 |  | 0.741 |
| Haptoglobin | 3868 |  | 397 | 1.086 (0.982, 1.199) | 0.107 |  | 528 | 1.046 (0.957, 1.143) | 0.326 |  | 0.585 |
| C3 | 4229 |  | 458 | 1.246 (1.145, 1.356) | <0.001 |  | 582 | 1.044 (0.957, 1.139) | 0.330 |  | 0.004 |
| CRP | 4332 |  | 476 | 1.151 (1.043, 1.271) | 0.005 |  | 590 | 1.096 (1.006, 1.194) | 0.036 |  | 0.458 |
| SuPAR | 4365 |  | 474 | 1.069 (0.969, 1.179) | 0.185 |  | 607 | 1.163 (1.076, 1.257) | <0.001 |  | 0.182 |
| *HR* hazard ratio, *CI* confidence interval, *NLR* neutrophil lymphocyte ratio, *CRP* C-reactive protein, *SuPAR* soluble urokinase plasminogen activator receptor | | | | | | | | | | | |
| ^a^ Adjusted hazard ratios and 95% confidence intervals, per 1 standard deviation (all such values) | | | | | | | | | | | |
| ^b^ Analysis by Cox proportional hazards model | | | | | | | | | | | |
| ^c^ *p* value associated with the null hypothesis that this variable has the same association with diabetes and cardiovascular disease, with all other effects being different; tests for all variables have 1 df | | | | | | | | | | | |
| ^d^ Adjusted for age, sex, waist circumference, smoking, systolic blood pressure, and anti-hypertensive drug medication | | | | | | | | | | | |
| ^e^ Adjusted for age, sex, waist circumference, smoking, systolic blood pressure, low-density lipoprotein, and anti-hypertensive drug medication | | | | | | | | | | | |
